# Multivariate Statistical Optimization of Tablet Formulations Incorporating High Doses of a Dry Herbal Extract

**DOI:** 10.3390/pharmaceutics11020079

**Published:** 2019-02-13

**Authors:** Euichaul Oh, Uijung Kim, Beom-Jin Lee, Cheol Moon

**Affiliations:** 1 College of Pharmacy, The Catholic University of Korea, Bucheon 14662, Korea; eoh@catholic.ac.kr (E.O.); ujkim84@naver.com (U.K.); 2 College of Pharmacy, Ajou University, Suwon 16499, Korea; bjl@ajou.ac.kr; 3 College of Pharmacy and Research Institute of Life and Pharmaceutical Sciences, Sunchon National University, Suncheon 57922, Korea

**Keywords:** herbal dry extract, herbal tablet formulation, multivariate statistical design, D-optimal mixture design, partial least square

## Abstract

The development of oral tablet formulation for herbal medicines has been restricted by large drug loadings and the poor physicochemical and mechanical properties of dry herbal extracts (DHEs). Herein, statistical experimental designs were applied to herbal tablet formulation development and optimization using Wuzi Yanzong dry extract (WYE). The tablet disintegration time and hardness were identified as the critical quality attributes (CQAs) of the product. The tablet formulation was designed to achieve a high drug loading (50% or higher of WYE), shorter tablet disintegration time (less than 30 minutes), and suitable hardness (6.0 to 7.5 kp). A D-optimal mixture design was used to evaluate the effects of excipients on CQAs to minimize the risk compression failure and improve the tabletability in formulations containing WYE at 50% and 65% by weight. A partial least squares model was used to elucidate the multivariate relationships between a large number of formulation variables and product CQAs, and determine the maximum possible WYE loading. From overlaid plots of the effects of formulation variables on CQAs, it was found that a maximum WYE loading of 67% in tablet formulation satisfied the acceptance criteria of CQAs. In conclusion, this study shows that multivariate statistical tools are useful for developing tablet formulations containing high doses of herbal extracts and establishing control strategies that ensure product quality.

## 1. Introduction

Herbal medicines are now well-accepted complementary and alternative medicines due to their unique advantages, which include few side effects, affordability, low cost, and widespread availability [1,2]. Traditional formulations of herbal medicines are commonly liquids derived from macerations, infusions, and decoctions, and have the shortcomings of large dose volumes, poor stabilities, and short storage lives. Therefore, there is a demand for solid oral dosage forms that are more stable and convenient [3]. Tablets are simple, convenient, and inexpensive oral solid dosage forms with high physicochemical and microbiological stabilities. However, the dry herbal extracts (DHEs), which are the main type of active pharmaceutical ingredients in herbal tablets, require high doses to achieve their desired effects, and high DHE loadings increase tablet disintegration time and retard drug release [4]. Furthermore, the unfavorable physicochemical and mechanical properties of DHEs, such as their hygroscopicity, stickiness, and bulkiness, cause the poor flowability and compressibility in tablet compression process [5]. Accordingly, the incorporation of large amounts of the DHEs into a tablet of acceptable size with appropriate disintegration time and hardness is a major challenge in the formulation of herbal tablets [4,6,7]. Some alternative formulations and processes have been proposed to cope with the unfavorable characteristics of DHEs, and may provide a means of addressing these issues [8]. For example, in general, the addition of disintegrants to the tablet formulation or high amount of magnesium stearate blended with the granules can promote the tablet integration, and the dry granulation of hygroscopic herbal extracts can improve their poor compressibility [9].

Scientific and systematic approaches in the development and optimization of formulations have been employed to understand drug products for the last decade [10,11]. Statistical experimental designs can be useful tools in pharmaceutical product development by providing information regarding correlations between independent variables or factors such as material attributes, process parameters, and dependent variables or response variables related to product quality attributes in an efficient and satisfactory way [12]. In particular, multivariate analysis can be used to understand the nature of such correlations effectively and comprehensively. Principal component analysis (PCA) and partial least squares (PLS) regression have been used to establish a model of the effects of raw material properties and process parameters on complicated formulations or manufacturing processes [13]. Although the quality of herbal tablets is influenced by numerous parameters during the formulation and manufacturing process, conventionally, herbal medicine formulations have been developed based on empirical trial-and-error approaches, thereby resulting in a high variability of the product quality. However, comparatively few reports have been issued on the implementation of statistical experimental designs in herbal medicine development or their use to understand more comprehensively the interrelations between formulation variables and product characteristics. [14]. 

In the present study, multivariate statistical models were used to design a herbal tablet formulation of dry Wuzi Yanzong extract (WYE) as a model for dry herbal extract, which is a traditional Chinese herbal formula that is said to replenish kidneys with vital essence, and is used for the treatment of male infertility, late onset hypogonadism, and kidney deficiency [15,16,17]. Due to its poor compressibility and hygroscopicity, the oral solid dosage forms of WYE are limited to teabags or pills. The aim of this study was to develop a herbal tablet formulation containing a high loading of WYE (50% or higher of WYE) with an acceptable disintegration time (less than 30 minutes) and hardness (6.0 to 7.5 kp). Target product characteristics such as dosage form, assay, content uniformity, disintegration time, and hardness were defined for the oral solid tablet formulation based on prior formulation knowledge. Statistical experimental designs, including a response surface model (D-optimal mixture design) and a multivariate model (PCA and PLS), were applied to WYE tablet formulation development and optimization.

## 2. Materials and Methods

### 2.1. Materials

The Wuzi Yanzong formula consists of five types of seeds: Cuscutae semen (*Cuscuta chinensis*), Lycii fructus (*Lycium barbarum*), Rubi fructus (*Rubus chingii*), Schizandrae fructus (*Schizandra chinensis*), and Plantaginis semen (*Plantago asiatica*) [16]. The herbal seeds were obtained from Ominherb (Deagu, Korea). Microcrystalline cellulose (MCC, Heweten^®^ 102, JRS Pharma, Rosenberg, Germany), croscarmellose sodium (CCS, Ac-Di-Sol^®^, FMC biopolymer, Philadelphia, PA, USA), crospovidone (Cros, Kollidone^®^ CL, BASF, Ludwigshafen, Germany), silicon dioxide (SiO_2_, Aerosil^®^-200, Evonic Degussa, Essen, Germany), and magnesium stearate (Mg St, FACI, Jurong Island, Singapore) were purchased from Whawon Pharm (Seoul, Korea). All of the other reagents were of analytical or of high-performance liquid chromatograpy (HPLC) grade.

### 2.2. Herbal Tablet Formulation

Wuzi Yanzong dry powder extract (WYE) was obtained by hot water extraction, evaporation under reduced pressure (R-300, Buchi, Flawil, Switzerland), and drying in a vacuum oven (SH-VDO-216NG, SH Scientific, Sejong, Korea). WYE was pretreated with Mg St and SiO_2_ to reduce its stickiness, and then blended with MCC, CCS, and Cros using a turbula mixer (DM-T2, Daemyung Enterprise, Kwangmyoung, Korea). Dry granules were obtained by blend compaction using a roller compactor (CCS220/M3B, Fitzpatrick, Waterloo, ON, Canada) under a hydraulic roll pressure of 75 bar. The lubricant, Mg St, was blended with dry granules and compressed at 35 kg/cm^2^ using a rotary tablet press (XP1, Korsch, Berlin, Germany).

### 2.3. Statistical Experimental Design

A D-optimal mixture design was used to evaluate the effects of excipients on disintegration time and hardness, in order to minimize the risk of tablet compression failure and improve the tabletabilities of formulations containing 50% or 65% WYE. Two different 21-run, five-factor, two-level D-optimal mixture designs were employed at the fixed WYE levels (50% and 65%). Amounts of CCS (X_1_), Cros (X_2_), MCC (X_3_), Mg St (X_4_), and SiO_2_ (X_5_) in formulations containing 50% WYE were chosen in ranges of 0–15%, 0–15%, 15–44.3%, 1.5–4%, and 1.5–6%, respectively, as shown in Table 1. Corresponding amounts in the 65% WYE formation were 0–10%, 0–10%, 20–31%, 2–4%, and 2–5%, respectively. Disintegration time and hardness were selected as responses, and the analysis was performed using Design-Expert^®^ software (version 8; Stat-Ease Inc., Minneapolis, MN, USA). Empirical mathematical equations describing the relationship between formulation ingredients and responses were prepared from the best-fit model.

Multivariate analysis was performed using SIMCA P+ 13.0 software (Umetrics Inc., Umea, Sweden) for the establishment of PCA and PLS regression models to elucidate the multivariate relationships between formulation variables and quality attributes, such as tablet hardness and disintegration time [18,19]. Along with the addition of WYE level as a new variable, a PLS model was used for the determination of maximum WYE loading and assessment of interactions between formulation variables. Prior to the analysis, all of the data were unit variance scaled. Amounts of WYE (three levels), CCS, Cros, MCC (12 levels), Mg St, and SiO_2_ in formulations were chosen for 53 batches in the ranges 35–65%, 1–24%, 1–30%, 18–45%, 1–5%, and 1–6%, respectively.

### 2.4. Evaluation of Herbal Tablet Formulation

Tablet appearances, assay, disintegration times, and hardness were evaluated. An Agilent 1260 Infinity HPLC system equipped with an Agilent variable wavelength detector and a quaternary pump (Santa Clara, CA, USA) was used to assay major ingredients. Tablets were dissolved in methanol by sonicating for five minutes, and filtered through a 0.22-μm nylon membrane filter. The mobile phase was composed of mixes of 0.05% aqueous trifluoroacetic acid (TFA, solvent A) and pure acetonitrile (solvent B). Gradient elution was performed at a flow rate of 1.0 mL/min as follows; a linear gradient from 20% to 50% solvent A over 20 minutes, a linear gradient from 50% to 100% solvent A for 30 minutes, and a linear gradient from 100% to 20% solvent A for 10 minutes followed by re-equilibration for 10 minutes.

The disintegration time of the tablets was determined using three randomly selected tablets and a disintegration tester (ZT320, Erweka GmbH, Heusenstamm, Germany) according to the Korean Pharmacopoeia, but without disks. Distilled water at 37 °C was used as the test medium. The hardness of three randomly selected tablets for each test formulation were determined using a hardness tester (TBH125, Erweka GmbH, Heusenstamm, Germany). 

## 3. Results and Discussion

### 3.1. Evaluation of Tablet Quality Attributes

The recent approach to pharmaceutical development, known as quality by design, is based on the understanding of products and processes. Critical quality attributes (CQAs) of herbal tablet products should be proposed and justified for the characterization of the product. In addition, all of the information required in clinical settings, including intended use, product dosage, expected product quality criteria, and treatment regimens, should be thoroughly reviewed based on prior knowledge and experience [20]. In particular, due to their complex ingredients and poor solubilities, herbal medicines require higher therapeutic doses than synthetic drugs [21], and thus, drug loading maximization is a critical issue. In the case of WYE, its oral dose is three to 4.5 gram/day [22]. In the present study, a drug loading of 50% or higher was chosen in 400-mg round, shallow, convex, uncoated tablets to achieve effective therapeutic efficacy with patient compliance. The problem posed by high doses of DHE is that it is hygroscopic, sticky, and brittle, which cause poor tabletability and a long disintegration time [23]. Thus, we consider that the pretreatment of WYE with the antiadherents, Mg St and SiO_2_, would improve powder properties by reducing stickiness. Generally, pretreatment with SiO_2_ and Mg St as lubricants or glidants and the granulation with disintegrants such as MCC, CCS, and Cros using roller compaction are able to improve the effect of high stickiness and hygroscopicity of DHE on tablet compression. The roller compaction process is known as an appropriate granulation method for hygroscopic dry herbal extracts [24]. In a preliminary study, increasing WYE drug loading significantly increased disintegration time and brittleness, and adversely affected compaction properties. Therefore, it is crucial to maintain the mechanical rigidity with fast disintegration in high WYE loading tablets. Disintegration time (less than 30 minutes) and hardness (6.0 to 7.5 kp) were selected as CQAs and controlled in WYE tablet formulation development and optimization. A preliminary study confirmed that assay, content uniformity, and chemical stability were acceptable and well-controlled. Since herbal extracts are complex mixtures of multiple constituents, the developments of dissolution methods are relatively complex [25,26], and thus, we considered tablet disintegration testing a good alternative to dissolution testing.

Regarding the excipients in herbal tablet formulations, the disintegrant amounts of CCS and Cros were found to be directly related to disintegration time. Fibrous CCS acts as a superdisintegrant because of its swelling, wicking, and strain recovery characteristics [27]. Furthermore, the capillary action and ability to recover from viscoelastic deformation make insoluble Cros act as an effective disintegrant for hygroscopic drugs [28]. Generally, high compression pressure and tablet hardness increase disintegration time. MCC acts as a filler and increases tablet hardness and improves flowability, compressibility, and compactability [29]. In addition, levels of Mg St and SiO_2_ are considered to affect tablet physical rigidity [24,30]. Considering these effects of formulation variables, statistical experiments, including D-optimal mixture design and PLS regression, were designed to optimize the tablet formulation of WYE. Process parameters were fixed during the course of these experiments.

### 3.2. Formulation Optimization Using D-optimal Mixture Design

Statistical experimental designs are often used to evaluate differences between tablet formulations. Mixture design is useful for optimizing tablet formulations [31]. In a D-optimal mixture design of the 50% WYE formulation, 21 experimental runs (18 batch runs and three center points) were conducted using various combinations of CCS (*X_1_*), Cros (*X_2_*), MCC (*X_3_*), Mg St (*X_4_*), and SiO_2_ (*X_5_*), and their effects on the response variables, disintegration time (*Y_1_*), and hardness (*Y_2_*) are provided in Table 2. Primary responses, tablet disintegration time, and hardness were analyzed using statistical software. The best-fitting model showed the statistical significance in model fitting with the insignificance in the lack-of-fit. For a WYE loading of 50%, as shown in Table 3, the reduced special cubic model was statistically significant in describing the disintegration time, whereas the quadratic model fit well with tablet hardness. The mathematical models of disintegration time and tablet hardness are provided by equations (1) and (2).

*Disintegration Time (Y_1_) = 0.34238X_1_ + 0.45318X_2_ + 0.84864X_3_ − 15.91125X_4_ − 2.34239X_5_* *+ 0.062955X_1_X_2_ + 9.76549×10^−4^X_1_X_3_ + 0.37591X_1_X_4_* *+ 0.044394X_1_X_5_ − 0.013977X_2_X_3_ + 0.36834X_2_X_4_ + 0.012109X_2_X_5_* *+ 0.39642X_3_X_4_ + 0.053652X_3_X_5_ + 0.30752X_4_X_5_* *− 5.51489×10^−4^X_1_X_2_X_3_,* (1)

*Hardness (Y_2_) = 0.086986X_1_ + 0.056358X_2_ + 0.20531X_3_ + 1.49955X_4_ − 1.04499X_5_* *− 4.20430×10^−4^X_1_X_2_ + 3.60975×10^−4^X_1_X_3_ − 0.033621X_1_X_4_ + 0.019548X_1_X_5_* *+ 1.92707×10^−3^X_2_X_3_ − 0.033377X_2_X_4_ + 0.020832X_2_X_5_ − 0.036365X_3_X_4_* *+ 0.019589X_3_X_5_ − 9.98355×10^−3^X_4_X_5_,* (2)

R-squared and predicted R-squared are the coefficient of determination and the proportion of variance predicted by a mathematical model, respectively. As shown in Table 3, both R-squared and predicted R-squared values were greater than 0.75, which suggests that these models predict disintegration time and hardness well. A *p*-value of > 0.05, or lack-of-fit test for the models, indicated that there is no evidence that the models do not fit the data well.

**Figure 1:**
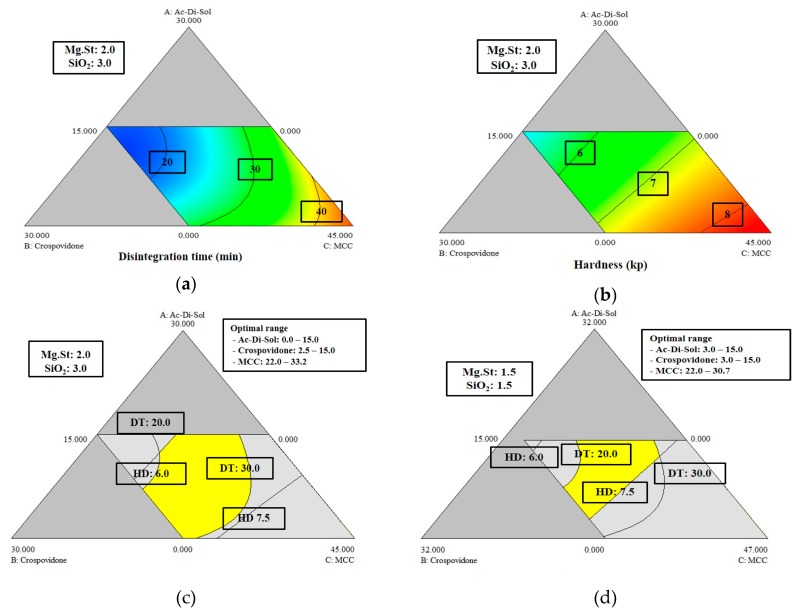
Contour diagrams of the relationships among the formulation variable levels, croscarmellose sodium (CCS, Ac-Di-Sol^®^), crospovidone (Cros), and microcrystalline cellulose (MCC) in fixed levels of magnesium stearate (Mg St, 2.0%) and silicon dioxide (SiO_2_, 3.0%) at the level of 50% of Wuzi Yanzong dry powder extract; (**a**) effects on disintegration time; (**b**) effects on hardness; (**c**) the overlaid diagrams of (**a**) and (**b**) for design space; and (**d**) overlaid diagrams of the effects on disintegration time and hardness in fixed level of magnesium stearate (Mg St, 1.5%) and silicon dioxide (SiO_2_, 1.5%). The yellow areas (design space) show the region where the best responses can be obtained. Figure (**a**) and (**b**) are modified triangular diagrams for three component systems. The number of each apex means the maximum level of each variable. The top corner point in (**a**) represents 30% of CCS (with 15% of MCC and 0% of Cros). The three lines (AB, BC, CA) joining the vertex points represent the combination of A, B, and C; they represent the two component or binary mixtures with the fixed level of the other variable, which is shown in the center on the line. For example, the levels of the variables at the center on line AB are: 15% of CCS, 15% of Cros, and 15% of MCC. The levels of the variables at the center of line BC are 0% of CCS, 15% of Cros, and 30% of MCC.

As shown in Figure 1a,b, higher levels of CCS or Cros reduced the disintegration time and increased the hardness, whereas higher MCC levels increased the disintegration time and decreased the hardness. The optimal levels of excipients in the formulation that were required to satisfy the acceptance criteria of the disintegration time and hardness were determined from overlaid diagrams of the effects of multidimensional combinations and interactions of formulation variables on disintegration time and hardness. The yellow area, which is also called the design space, in Figure 1c,d represent the region where the best responses were obtained at fixed Mg St and SiO_2_ levels at a WYE level of 50%. Since the size of the design space depends on the levels of fixed excipients, the design space can be changed by changing the Mg St and SiO_2_ levels. To verify established design spaces, five random points were selected, and tablets were prepared accordingly; the disintegration time and hardness values of these tablets were found to fully satisfy the acceptance criteria (Table 4).

Similar to the experimental design for 50% WYE formulations, in the experimental design of the 65% WYE formulation, 21 experimental runs (18 batch runs and three center points) were conducted using various combinations of CCS (*X_1_*), Cros (*X_2_*), MCC (*X_3_*), Mg St (*X_4_*), and SiO_2_ (*X_5_*), and their effects on the response variables, disintegration time (*Y_1_*), and hardness (*Y_2_*), are summarized in Table 5. The reduced special cubic model was statistically significant in describing the disintegration time, whereas the quadratic model was fit well in tablet hardness. Mathematical models for the disintegration time and hardness were provided by equations (3) and (4).

*Disintegration Time (Y_1_) = 0.31647X_1_ − 0.23514X_2_ + 1.28355X_3_ + 1.62077X_4_ − 2.23277X_5_* *+ 1.39135X_1_X_2_ + 0.028424X_1_X_3_ − 0.049960X_1_X_4_* *+ 0.013923X_2_X_3_ + 0.049232X_2_X_5_ + 0.049465X_3_X_5_* *− 0.077349X_1_X_2_X_3_,* (3)

*Hardness (Y_2_) = 0.16516X_1_ + 0.093511X_2_ + 0.28410X_3_ + 0.98091X_4_ − 0.97662X_5_* *− 7.99739×10^−4^X_1_X_2_ − 4.51043×10^−5^X_1_X_3_ − 0.039370X_1_X_4_ + 0.022078X_1_X_5_* *+ 3.16603×10^−3^X_2_X_3_ − 0.026998X_2_X_4_ + 0.022847X_2_X_5_ − 0.039099X_3_X_4_* *+ 0.024016X_3_X_5_ − 0.019296X_4_X_5_,* (4)

**Figure 2:**
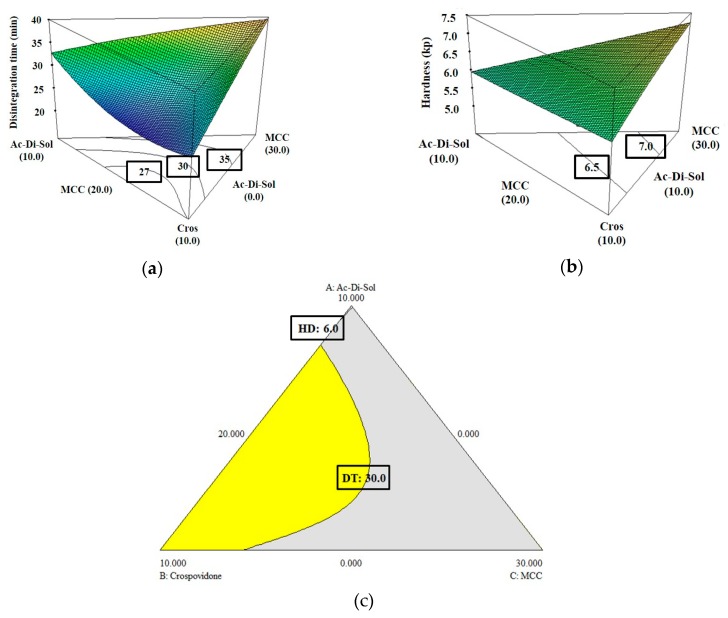
Response surface plots of the relationships among the formulation variable levels, croscarmellose sodium (CCS, Ac-Di-Sol^®^), crospovidone (Cros), and microcrystalline cellulose (MCC) in fixed level of magnesium stearate (Mg St, 2.0%) and silicon dioxide (SiO_2_, 3.0%) at the level of 65% Wuzi Yanzong dry powder extract; (**a**) effects on disintegration time; (**b**) effects on hardness; and (**c**) the overlaid contour diagram of (**a**) and (**b**) for the design space. The yellow areas (design space) show the region where the best responses can be obtained.

As shown in Table 6, the R-squared and predicted R-squared values indicated that these models fit well in the prediction for disintegration time and hardness. As shown in Figure 2a,b, higher levels of CCS or MCC increased the disintegration time, whereas higher Cros levels decreased the disintegration time, which suggests that insoluble Cros is a more effective disintegrant than CCS in formulations containing high levels of hygroscopic WYE. On the other hand, higher levels of CCS or Cros decreased tablet hardness. The effect of MCC on disintegration time and hardness was greater than that of Cros or CCS. 

Similar to the establishment of a design space to determine levels of excipients for formulations containing 50% WYE, in the design space for 65% WYE formulations, levels of Mg St and SiO_2_ were fixed at levels that are commonly used. In contrast to the 50% WYE design space, the overlaid yellow region for optimal levels of CCS, Cros, and MCC at fixed levels of Mg St and SiO_2_ was narrow in the 65% WYE design space (Figure 2c). To verify the established design spaces, five random points were selected in the design space, and we then confirmed that the disintegration time and hardness values of the tablets prepared using formulations corresponding to these points satisfied the acceptance criteria (Table 7). We found experimental designs using the D-optimal mixture design procedure provided models that predicted the effects of formulation variables on CQAs in tablets containing 50% or 65% WYE well.

### 3.3. Formulation Optimization Using Partial Least Squares Model

**Figure 3:**
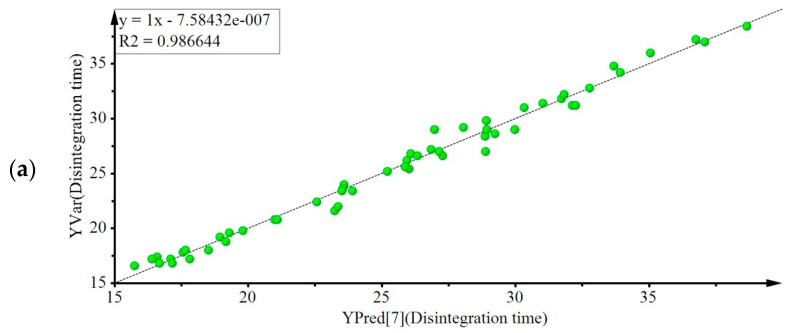

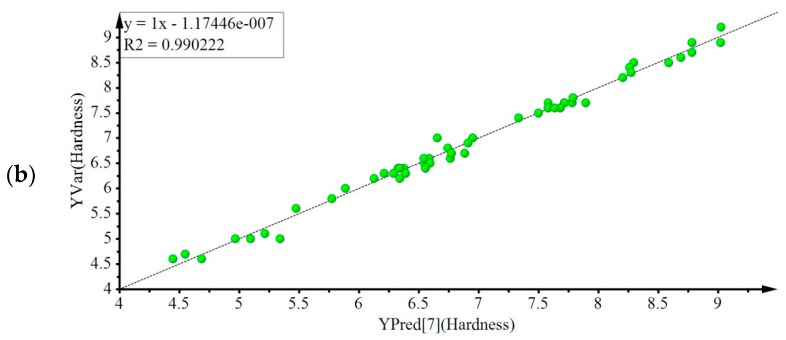
Predicted vs. measured plots in partial least squares model for (**a**) disintegration time; (**b**) hardness

Although the 65% WYE formulations with appropriate disintegration time and hardness values were verified using the D-optimal mixture design, the maximally loaded WYE formulations can be predicted using statistical designs that address more complex systems. Multivariate analysis methods such as PLS can deal with a large number of variables simultaneously, and thus can provide information that is not provided by D-optimal design methods [32]. PLS with 53 experimental runs and 21 components (six independent components, six squared independent components, and 15 interaction components) was used to establish a design space to elucidate the relationships between six formulation variables—that is, WYE, CCS, Cros, MCC, Mg St, and SiO_2_ levels—and the two response variables: disintegration time and hardness (Table 8). Cumulative R2 and Q2 values for the Y matrix in the seven component PLS regression model indicated a good model fit (R2) and good predictability for new data (Q2) (Table 9). Figure 3 shows the predicted versus measured plots for the disintegration time and hardness that were obtained using the calibration model. High R2 values and slopes indicated a good model fit. 

**Figure 4:**
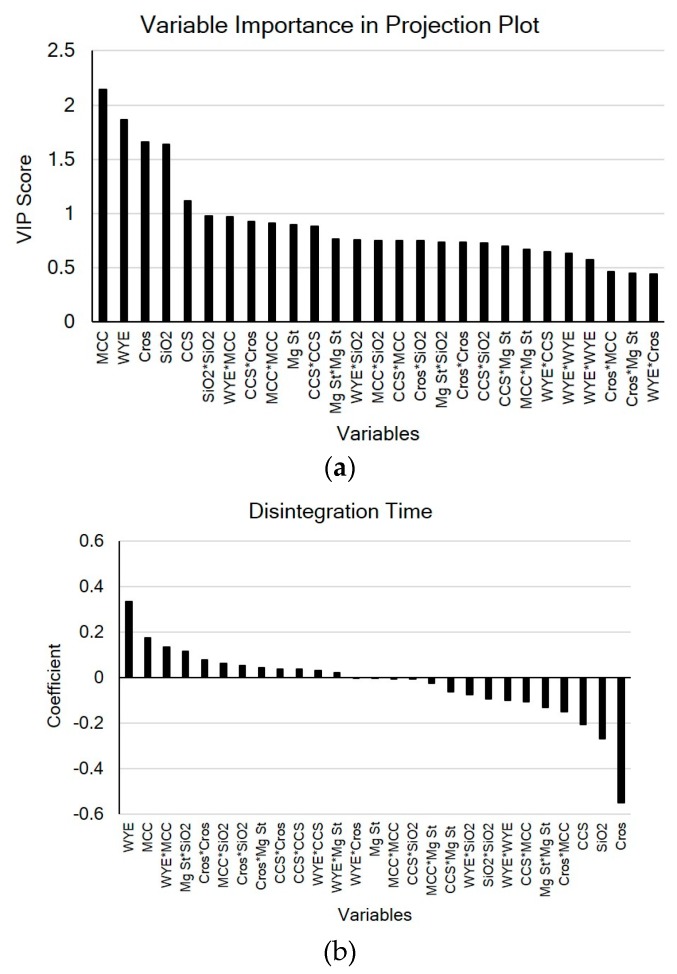

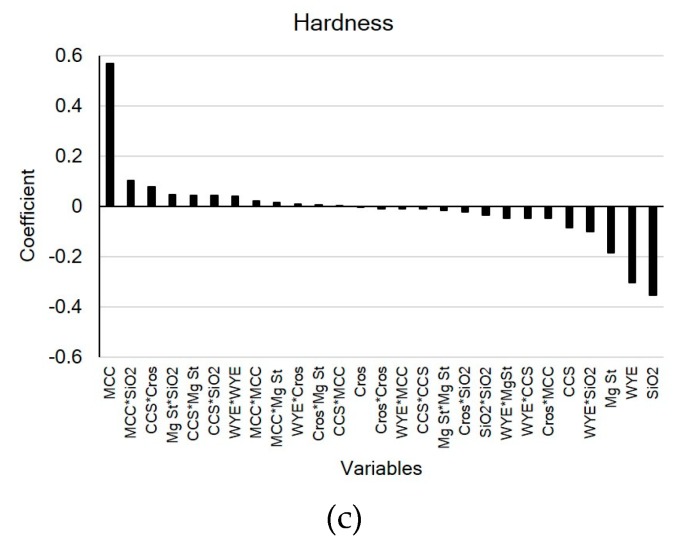
The variable importance in projection (VIP) plot (**a**) displaying important variables in terms of disintegration time and hardness; and coefficient plots for (**b**) disintegration time; and (**c**) hardness.

**Figure 5:**
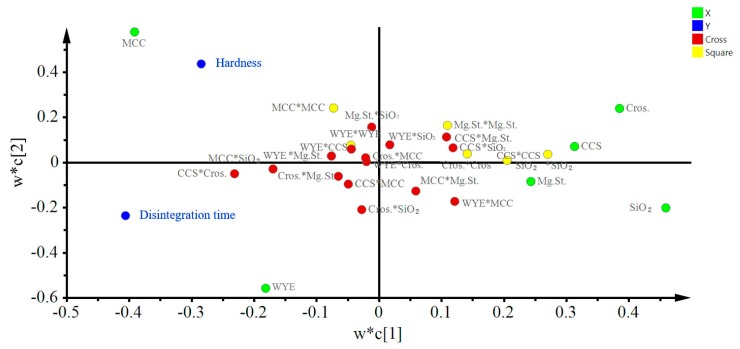
Loading plot displaying the relationships among variables and critical quality attributes (CQAs).

The variable importance in projection (VIP) in Figure 4a shows important model variables and that levels of MCC, WYE, Cros, and SiO_2_ are important with respect to disintegration time and hardness, whereas the coefficient plot shows that Cros and MCC are the most important variables with respect to disintegration time and hardness, respectively (Figure 4b,c). As shown in Figure 4b,c, the WYE level was positively correlated with the disintegration time and negatively correlated with the hardness, which coincides with a preliminary study on the mechanical properties of WYE. The loading plot in Figure 5 shows relationships between all variables and two CQAs: disintegration time and hardness. In the figure, variables near each other (e.g., MCC and hardness) are positively correlated, whereas variables that are opposite from the origin (e.g. Cros and disintegration time) are negatively correlated. 

**Figure 6:**
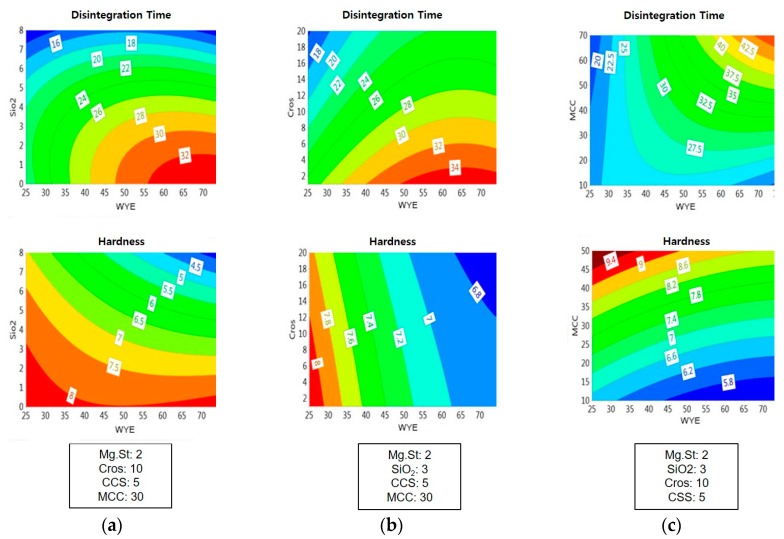
Contour plots depicting the effects of (**a**) silicon dioxide (SiO_2_) vs. Wuzi Yanzong dry extract (WYE); (**b**) Crospovidone (Cros) vs. Wuzi Yanzong dry extract (WYE); and (**c**) microcrystalline cellulose (MCC) vs. Wuzi Yanzong dry extract (WYE) on disintegration time (top) and hardness (bottom).

The effects of variables and their interactions on responses are schematized in the contour plots shown in Figure 6. For example, when variables were fixed at 5% CCS, 10% Cros, 50% MCC, and 2% Mg St, a higher level of WYE or a lower SiO_2_ level resulted in greater disintegration times. In contrast, higher levels of WYE and SiO_2_ resulted in a lower hardness; and the effect of SiO_2_ on hardness was greater than that of WYE, as shown in Figure 6a. The effect of Cros on disintegration time was greater than that of WYE, whereas its effect on hardness was much less than that of WYE (Figure 6b). A complex relationship was observed between MCC and WYE regarding hardness, as shown in Figure 6c. At a lower MCC levels (<10%), the effect of the WYE level on disintegration time was only slight, but at higher MCC levels (>40%), the effect of the WYE level on disintegration time was marked.

**Figure 7:**
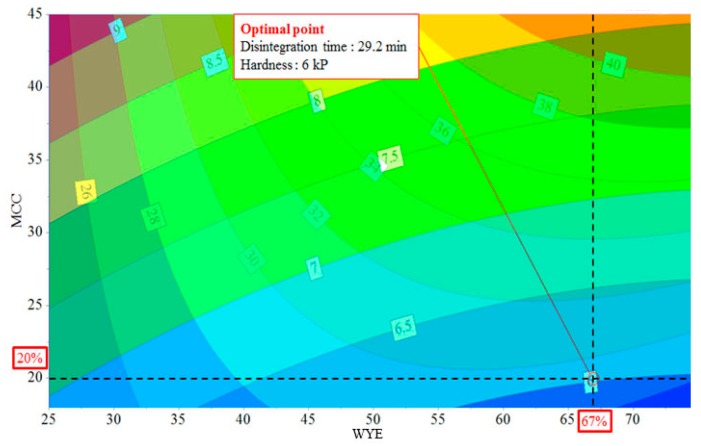
Overlaid response contour plot for the effects of microcrystalline cellulose (MCC) vs. Wuzi Yanzong dry extract (WYE) on disintegration time and hardness.

From the overlaid plots of the effects of MCC vs. WYE on disintegration time and hardness, we found the formulation with the highest WYE loading formulation that satisfied CQA acceptance criteria (Figure 7); that is, the formulation containing 67% WYE and 20% MCC, which had a disintegration time of 29.2 minutes and a hardness of six kp. In summary, multivariate statistical tools, including the response surface model and PLS, were found to be useful for optimizing formulations, evaluating the effects of formulation variables and their interactions on CQAs in herbal tablets containing high amounts of dry herbal extract, and understanding these relations.

## 4. Conclusions

This study demonstrates the effectiveness of statistical tools on elucidating relationships between formulation variables and herbal tablet quality attributes. D-optimal mixture design and the PLS model were found to be highly predictable statistical models in terms of evaluating the effects of formulation variables on CQAs. Furthermore, the optimized formulation containing the maximum WYE loading fully met the CQA acceptance criteria and had good tabletability. We believe that our findings will be useful for developing tablet formulations containing high levels of herbal extract and establishing control strategies to ensure product quality.

## Figures and Tables

**Table 1:** Independent variables (percent composition of ingredients) of the experimental designs for Wuzi Yanzong extract tablet formulation.

Experimental Design	D-optimal Mixture Design				Partial Least Square	
Independent Variables	Low Level	High Level	Low Level	High Level	Low Level	High Level
Wuzi Yanzong extract	50	50	65	65	35	65
Croscarmellose sodium	0	15	0	10	1	24
Crospovidone	0	15	0	10	1	30
Microcrystalline cellulose	15	44.3	20	31	18	45
Magnesium stearate	1.5	4	2	4	1	5
Silicon dioxide	1.5	6	2	5	1	6

**Table 2:** Experimental design of herbal tablet formulation containing 50% of Wuzi Yanzong dry powder extract with five control factors (%, *w*/*w*) and the resulting responses, disintegration time, and hardness.

Run	CCS (%)	Cros. (%)	MCC (%)	Mg St (%)	SiO_2_ (%)	Disintegration Time (min)	Hardness (kp)
1	7.4	7.4	28.6	2.7	3.7	26.6 ± 1.3	6.6 ± 0.2
2	0.0	15.0	27.1	1.9	6.0	24.0 ± 1.5	5.9 ± 0.3
3	8.7	6.5	26.8	2.6	5.4	24.6 ± 1.2	5.8 ± 0.4
4	15.0	12.9	15.6	4.0	2.4	21.2 ± 0.9	5.1 ± 0.4
5	7.4	7.4	28.6	2.7	3.7	26.6 ± 1.8	6.5 ± 0.7
6	0.0	3.1	36.9	4.0	6.0	36.0 ± 2.1	6.4 ± 0.2
7	0.0	15.0	28.4	4.0	2.6	30.0 ± 1.8	6.8 ± 0.5
8	13.4	15.0	17.3	2.7	1.6	20.6 ± 1.1	5.6 ± 0.4
9	15.0	0.0	31.8	1.6	1.6	37.2 ± 2.3	7.5 ± 0.6
10	7.4	7.4	28.6	2.7	3.7	26.8 ± 1.7	6.4 ± 0.5
11	7.2	15.0	17.8	4.0	6.0	16.6 ± 1.0	4.4 ± 0.3
12	11.7	2.1	29.3	1.5	5.4	29.4 ± 0.8	6.2 ± 0.1
13	14.9	14.9	15.0	1.5	3.6	18.2 ± 0.9	5.0 ± 0.0
14	0.0	0.0	44.3	4.0	1.7	46.4 ± 2.6	8.3 ± 0.4
15	5.6	11.0	30.4	1.5	1.5	25.2 ± 1.2	7.6 ± 0.2
16	8.4	12.7	21.4	1.5	6.0	16.8 ± 1.4	5.1 ± 0.5
17	0.0	11.3	35.7	1.5	1.5	33.8 ± 1.7	8.3 ± 0.7
18	6.9	2.7	33.8	2.8	3.7	34.4 ± 2.5	7.0 ± 0.6
19	15.0	10.9	15.4	2.7	6.0	17.6 ± 0.7	4.1 ± 0.1
20	15.0	1.1	24.0	4.0	6.0	28.4 ± 1.4	5.0 ± 0.2
21	0.0	0.0	42.7	1.5	5.8	40.2 ± 2.0	7.4 ± 0.4

Disintegration time and hardness were tested in triplicate (Mean ± SD, n = 3). Abbreviation: CCS, croscarmellose sodium (Ac-Di-Sol^®^); Cros, crospovidone; MCC, microcrystalline cellulose; Mg St, magnesium stearate; SiO_2_, silicon dioxide.

**Table 3:** Summary statistics for reduced special cubic model for disintegration time and quadratic model for hardness in herbal tablets containing 50% of Wuzi Yanzong dry powder extract.

Model Statistics	Disintegration Time (*Y_1_*)	Hardness (*Y_2_*)
Model, *p*-value	<0.0001	<0.0001
R2	0.9999	0.9991
Adjust R2	0.9995	0.9971
Predicted R2	0.9868	0.9876
Standard deviation	0.18	0.064
PRESS	17.30	0.36
Lack of fit, *p*-value	0.2251	0.9613

PRESS, Predicted residual sum of squares; *p* < 0.05 is significant.

**Table 4:** Optimal points obtained from the design spaces for Wuzi Yanzong extract tablet formulation containing 50% Wuzi Yanzong dry powder extract and the resulting response, disintegration time, and hardness.

No.	CCS (%)	Cros (%)	MCC (%)	Mg St. (%)	SiO_2_ (%)	Disintegration Time (min)	Hardness (kp)
1	10.0	15.0	22.0	1.5	1.5	18.8	6.5
2	11.3	13.7	22.0	1.5	1.5	18.9	6.5
3	8.2	15.0	23.0	1.5	2.3	19.2	6.5
4	2.6	14.5	27.1	1.5	4.3	22.8	6.5
5	5.3	15.0	24.7	2.9	2.1	23.4	6.5

Abbreviation: CCS, croscarmellose sodium (Ac-Di-Sol^®^); Cros, crospovidone; MCC, microcrystalline cellulose; Mg St, magnesium stearate; SiO_2_, silicon dioxide.

**Table 5:** Experimental design of herbal tablet formulation containing 65% Wuzi Yanzong dry powder extract with five control factors (%, *w*/*w*) and the resulting response, disintegration time, and hardness.

Run	CCS (%)	Cros (%)	MCC (%)	Mg St. (%)	SiO_2_ (%)	Disintegration Time (min)	Hardness (kp)
1	7.4	3.6	20.0	2.0	2.0	28.9 ± 1.4	6.5 ± 0.3
2	0.0	9.7	20.0	3.3	2.0	29.9 ± 1.2	6.3 ± 0.2
3	0.0	0.0	28.6	2.0	4.4	36.5 ± 1.9	6.4 ± 0.5
4	0.0	0.0	26.0	4.0	5.0	34.9 ± 2.1	5.1 ± 0.2
5	5.7	0.0	25.3	2.0	2.0	39.1 ± 1.8	7.1 ± 0.4
6	0.0	10.0	20.0	2.0	3.0	27.2 ± 1.0	6.2 ± 0.1
7	0.0	0.0	28.6	4.0	2.4	41.1 ± 2.8	6.7 ± 0.1
8	0.0	6.6	20.1	4.0	4.3	28.9 ± 0.5	5.0 ± 0.2
9	10.0	0.0	20.0	2.4	2.6	33.7 ± 1.3	6.0 ± 0.5
10	0.0	6.9	20.7	2.3	5.0	26.2 ± 0.6	5.1 ± 0.4
11	8.0	0.0	20.0	2.0	5.0	29.2 ± 1.2	5.0 ± 0.3
12	9.0	0.0	20.0	4.0	2.0	35.9 ± 1.0	5.8 ± 0.5
13	0.0	4.8	26.2	2.0	2.0	36.1 ± 1.7	7.4 ± 0.5
14	5.0	3.2	20.0	3.1	3.8	27.8 ± 1.8	5.3 ± 0.3
15	0.0	0.0	31.0	2.0	2.0	41.7 ± 1.3	7.8 ± 0.6
16	2.6	5.6	22.6	2.0	2.2	27.6 ± 1.6	6.8 ± 0.5
17	2.6	1.8	23.4	4.0	3.3	33.5 ± 1.2	5.7 ± 0.4
18	3.4	6.1	20.0	2.3	3.2	25.7 ± 0.4	5.9 ± 0.1
19	2.9	2.9	22.9	2.9	3.3	30.5 ± 2.5	6.0 ± 0.4
20	2.9	2.9	22.9	2.9	3.3	30.6 ± 1.8	5.9 ± 0.3
21	2.9	2.9	22.9	2.9	3.3	30.4 ± 1.1	6.1 ± 0.3

Disintegration time and hardness were tested in triplicate (Mean ± SD, n = 3). Abbreviation: CCS, croscarmellose sodium (Ac-Di-Sol®); Cros, crospovidone; MCC, microcrystalline cellulose; Mg St, magnesium stearate; SiO_2_, silicon dioxide.

**Table 6:** Summary statistics for a reduced special cubic model for disintegration time and quadratic model for hardness in herbal tablets containing 65% Wuzi Yanzong dry powder extract.

Model Statistics	Disintegration Time (Y_1_)	Hardness (Y_2_)
Model, *p*-value	<0.0001	<0.0001
R2	0.9980	0.9978
Adjust R2	0.9956	0.9926
Predicted R2	0.9419	0.9411
Standard deviation	0.32	0.067
PRESS	26.87	0.72
Lack of fit, *p*-value	0.0691	0.9310

PRESS, Predicted residual sum of squares; *p* < 0.05 is significant.

**Table 7:** Optimal points obtained from design spaces for Wuzi Yanzong extract tablet formulation containing 65% Wuzi Yanzong dry powder extract and the resulting response, disintegration time, and hardness.

No.	CCS (%)	Cros (%)	MCC (%)	Mg St. (%)	SiO_2_ (%)	Disintegration Time (min)	Hardness (kp)
1	3.4	7.2	20.2	2.0	2.2	26.0	6.5
2	3.3	7.6	20.0	2.0	2.1	26.1	6.5
3	3.7	7.2	20.0	2.0	2.1	26.1	6.5
4	3.5	6.6	20.6	2.0	2.3	26.1	6.5
5	2.2	8.6	20.0	2.0	2.2	26.3	6.5

Abbreviation: CCS, croscarmellose sodium (Ac-Di-Sol^®^); Cros, crospovidone; MCC, microcrystalline cellulose; Mg St, magnesium stearate; SiO_2_, silicon dioxide.

**Table 8:** Experimental design of Wuzi Yanzong extract tablet formulation with control factors (%, *w*/*w*) and the resulting response, disintegration time, and hardness for partial least squares (PLS) regression.

WYE (%)	CCS (%)	Cros (%)	MCC (%)	Mg St. (%)	SiO_2_ (%)	Disintegration Time (min)	Hardness (Kp)
50	2	5	40	1.5	1.5	37.0 ± 1.6	8.6 ± 0.8
50	5	2	40	1.5	1.5	38.4 ± 1.5	8.5 ± 0.3
50	3	9	34	1	3	29.8 ± 1.2	7.7 ± 0.5
50	3	9	34	3	1	31.8 ± 0.7	7.8 ± 0.4
50	6	6	34	1	3	31.0 ± 1.1	7.7 ± 0.6
50	6	6	34	3	1	32.8 ± 1.0	7.7 ± 0.2
50	9	3	34	1	3	32.2 ± 1.3	7.6 ± 0.5
50	9	3	34	3	1	34.2 ± 1.5	7.7 ± 0.1
50	2	16	26	2	4	24.0 ± 0.4	6.4 ± 0.0
50	2	16	26	4	2	26.2 ± 1.2	6.6 ± 0.4
50	2	16	26	3	3	25.4 ± 1.6	6.5 ± 0.2
50	8	10	26	2	4	25.2 ± 1.1	6.3 ± 0.3
50	8	10	26	4	2	27.2 ± 1.3	6.5 ± 0.3
50	8	10	26	3	3	26.6 ± 1.8	6.4 ± 0.3
50	14	4	26	2	4	27.0 ± 1.3	6.2 ± 0.6
50	14	4	26	4	2	29.2 ± 1.9	6.4 ± 0.1
50	14	4	26	3	3	28.4 ± 1.4	6.3 ± 0.3
50	3	21	18	4	4	21.6 ± 1.1	5.1 ± 0.2
50	3	21	18	2	6	17.4 ± 0.5	4.7 ± 0.0
50	10	14	18	4	4	22.0 ± 1.1	5.0 ± 0.7
50	10	14	18	2	6	17.8 ± 0.6	4.6 ± 0.1
50	17	7	18	4	4	23.4 ± 1.9	5.0 ± 0.3
50	17	7	18	2	6	19.2 ± 2.0	4.6 ± 0.2
65	1	2	28	2	2	37.2 ± 1.5	7.5 ± 0.3
65	1	3	26	3	2	36.0 ± 1.8	7.0 ± 0.5
65	3	1	26	2	3	34.8 ± 1.5	6.8 ± 0.4
65	2	5	23	3	2	31.2 ± 1.9	7.0 ± 0.4
65	4	4	23	2	2	31.2 ± 1.6	6.9 ± 0.7
65	5	2	23	2	3	31.4 ± 1.2	6.4 ± 0.3
65	3	8	20	2	2	27.0 ± 1.1	6.6 ± 0.6
65	4	6	20	3	2	29.0 ± 1.8	6.2 ± 0.3
65	5	4	20	3	3	29.0 ± 1.3	5.8 ± 0.8
65	6	3	20	2	4	26.8 ± 1.8	5.6 ± 0.2
65	4	3	20	4	4	29.0 ± 1.1	5.0 ± 0.8
65	8	2	20	2	3	28.6 ± 0.3	6.0 ± 0.3
35	3	15	45	1	1	20.8 ± 0.7	9.2 ± 0.6
35	6	10	45	3	1	23.6 ± 1.0	8.9 ± 0.5
35	4	8	45	4	4	26.6 ± 1.5	8.7 ± 0.8
35	11	5	45	1	3	25.6 ± 1.3	8.9 ± 0.7
35	4	18	39	2	2	19.8 ± 1.1	8.5 ± 0.1
35	7	14	39	3	2	20.8 ± 0.8	8.4 ± 0.8
35	10	10	39	3	3	22.4 ± 1.6	8.3 ± 0.3
35	15	5	39	2	4	23.4 ± 1.2	8.2 ± 0.9
35	5	25	32	2	1	16.8 ± 0.8	7.7 ± 0.2
35	8	20	32	2	3	18.0 ± 0.9	7.6 ± 0.6
35	10	18	32	1	4	17.2 ± 1.1	7.6 ± 0.8
35	16	12	32	4	1	18.0 ± 0.8	7.6 ± 0.4
35	14	10	32	4	5	19.6 ± 0.9	7.4 ± 0.5
35	5	30	24	3	3	18.8 ± 1.4	6.7 ± 0.4
35	13	20	24	5	3	17.2 ± 1.0	6.7 ± 0.5
35	15	16	24	5	5	16.8 ± 0.7	6.6 ± 0.6
35	20	10	24	5	6	16.6 ± 0.6	6.4 ± 0.3
35	24	8	24	3	6	17.2 ± 0.3	6.3 ± 0.4

Disintegration time and hardness were tested in triplicate (mean ± SD, n = 3). Abbreviation: WYE, Wuzi Yanzong dry powder extract; CCS, croscarmellose sodium (Ac-Di-Sol^®^); Cros, crospovidone; MCC, microcrystalline cellulose; Mg St, magnesium stearate; SiO_2_, silicon dioxide.

**Table 9:** The diagnostics for partial least squares (PLS) regression model.

Principal Component	R2Y (Cumulation)	Q2Y (Cumulation)
Component [1]	0.4053116	0.332277
Component [2]	0.738942	0.676162
Component [3]	0.860685	0.793175
Component [4]	0.9214	0.84413
Component [5]	0.96235	0.895763
Component [6]	0.977945	0.913375
Component [7]	0.988433	0.928783
